# Genetic analysis of the maximum drinks phenotype

**DOI:** 10.1186/1471-2156-6-S1-S124

**Published:** 2005-12-30

**Authors:** Scott F Saccone, Nancy L Saccone, Rosalind J Neuman, John P Rice

**Affiliations:** 1 Department of Psychiatry, Washington University School of Medicine, St. Louis, Missouri 63110 USA; 2 Department of Genetics, Washington University School of Medicine, St. Louis, Missouri 63110 USA

## Abstract

Using data provided by the Collaborative Study on the Genetics of Alcoholism we studied the genetics of a quantitative trait: the maximum number of drinks consumed in a 24-hour period. A two-stage method was used. First, linkage analysis was performed, followed by association analysis in regions where linkage was detected. Additionally, the extent of linkage disequilibrium among single-nucleotide polymorphisms (SNP) associated with the phenotype was assessed. Linkage to chromosomes 2 and 7 was detected, and follow-up association analysis found multiple trait-associated SNPs in the chromosome 7 linkage region. Chromosome 4, which has been implicated in previous studies of the maximum drinks phenotype, did not pass our threshold for linkage evidence in stage 1, but secondary analyses of this chromosome indicated modest evidence for both linkage and association. The evidence suggests that chromosome 7 may harbor an additional locus influencing the maximum drinks consumption phenotype.

## Background

The data provided by the Collaborative Study on the Genetics of Alcoholism (COGA) for Genetic Analysis Workshop 14 (GAW14) includes the "maximum number of drinks consumed in a 24-hour period." This phenotype is closely related to alcoholism diagnosis, and a previous genome screen of this phenotype in COGA resulted in evidence for linkage to chromosome 4 in the vicinity of the alcohol dehydrogenase (ADH) gene cluster [1]. The GAW14 dataset provides nearly 16,000 genotyped single-nucleotide polymorphisms (SNP) that were not available in the original COGA data. For this report we have analyzed these additional genotypic data to see whether the original linkage findings can be confirmed or extended. Linkage signals were followed up with association analysis to make use of the density of the SNP data to refine linkage signals and potentially localize genetic variants that may influence alcohol consumption.

## Methods

In defining the quantitative trait, we set the phenotype of individuals who report a maximum drinks value of zero to be unknown, since these unexposed individuals have undetermined response to alcohol. We reduced skewness by taking the logarithm of the maximum number of drinks. A linear regression was used to correct for sex, and the final trait value was defined as the residual from the regression.

Our primary analysis consisted of two stages, applied to the cleaned SNP data. In the first stage we tested for genetic linkage using the SNP marker data provided by Illumina. Nonparametric two-point quantitative trait linkage analysis was performed using the "--qtl" option of the program MERLIN [1]. The method compares allele sharing among individuals with similar phenotypes; individuals at extreme ends of the distribution are given greater weight. We chose two-point rather than multipoint analysis because strong linkage disequilibrium (LD) among the densely spaced SNP markers could lead to inflated evidence for linkage if multipoint methods are used without accounting for marker-marker LD. Markers exhibiting linkage with a LOD score greater than 1.8 were used to define regions for further study. In the second stage a 20-cM interval centered at the marker was used to test for trait association. SNPs in this interval from the combined Illumina and Affymetrix sets were analyzed for trait association using the quantitative pedigree disequilibrium test (QPDT) [2] as implemented in the program UNPHASED [3]. Our choice to use the Illumina SNP set (4,710 markers) for the first stage and the Affymetrix SNP set (11,120 markers) together with the Illumina set for the follow-up association analysis allowed us to carry out the initial genome screen and then proceed with fine-mapping in regions of linkage using the more densely spaced SNPs provided by Affymetrix.

To supplement our primary two-stage analysis, we carried out some related additional analyses. These are described below.

For comparison with the stage 1 SNP linkage screen, linkage analysis of the autosomal microsatellite data was performed using the same two-point nonparametric analysis as above. We also repeated this microsatellite analysis using a 2-cM multipoint grid because LD among these markers was not a significant concern.

For chromosome 4, prior linkage evidence for the maximum drinks phenotype has been reported [1], making this a chromosome of special interest. Using the Illumina markers for chromosome 4, we then tested for linkage in the first stage using a regression of the estimated IBD status against the squared sum and squared difference of the trait values, as implemented by the program MERLIN-REGRESS [4]. As this analysis method is computationally intensive, we applied it only to chromosome 4, allowing a more comprehensive study of this chromosome of interest.

LD between markers should be considered when using SNP data to search for disease or trait association. Thus, SNPs exhibiting trait association in our primary analysis that were also in close proximity to one another were tested to see if they were in LD with each other. Pair-wise LD coefficients were computed using the program LDMAX, which is part of the GOLD computer package [5]; results are reported using the normalized coefficient *D' *= *D*/|*D*|_*max*_, where *D *= p_11 _- *p*_1 _*p*_2_, *p*_11 _is the frequency for the haplotype containing allele 1 at both markers, *p*_*i *_is the frequency of allele 1 at the *i*^th ^marker of the pair, and |*D*|_*max *_is the maximum possible (absolute) value of D given the marker allele frequencies.

## Results

Out of a sample of 1,614 individuals, the maximum number of drinks was recorded for 1,388 (86%) individuals. The mean, median, and mode were 17.8, 12, and 24, respectively, with a standard deviation of 17.3. Due to computer memory constraints, 9 out of 143 families (6%), consisting of 243/1,614 (15%) individuals, were automatically dropped by MERLIN from the linkage analysis in stage 1, and this reduction in sample is a limitation of our analyses. The average maximum drinks value in the dropped families was slightly lower than the overall average (by 0.16 of a standard deviation); this difference does not appear to have a systematic cause and is not expected to affect the interpretability of the results.

We used 4,710 markers from the Illumina SNP set. The mean spacing between markers was 0.77 cM on the genetic map and 621 kb on the physical map, and the mean minor allele frequency (MAF) was 0.39. We used 11,120 markers from the Affymetrix SNP set. The mean spacing was 0.32 cM (258 kb on the physical map), and the mean MAF was 0.27.

In our primary analysis, only chromosomes 2 and 7 were found to have LOD scores over 1.8 at stage 1. Tables 1 and 2 show the results of the combined two-point linkage and trait association analysis (QPDT) for these two chromosomes. For the stage 2 association analyses, the tables report only those SNPs significant at the 0.01 level. We note that while multiple SNPs throughout the linked regions were significant at the 0.05 level, only one SNP on chromosome 2 and multiple SNPs on chromosome 7 gave association results significant at the 0.01 level. Table 3 lists the results for the additional two-point regression analysis performed on chromosome 4 by MERLIN-REGRESS and the QPDT results computed by UNPHASED for SNPs in the linkage regions detected by the regression. The maximal LOD of 2.14 occurred at 97.4 Mb (100.4 cM).

We computed values of *D*' for the significantly associated SNPs on chromosomes 2 and 7 (Tables 1 and 2). Thus, we focused on chromosome 7 because multiple significant SNPs were detected there. An LD block extended from 124.757 Mb to 124.791 Mb, covering the three most significantly associated SNPs; in fact, this block satisfied |*D*'| ≥ 0.9 for all marker pairs. Thus, the evidence for association in this region does not come from three independent SNPs but rather indicates a single LD block associated with the phenotype.

Our analyses of the microsatellite data found no evidence for linkage satisfying our LOD threshold of 1.8 in either the two-point or the multipoint screens. For the two-point screen, the only LOD scores over 1.0 occurred on chromosome 7 at D7S821 (LOD = 1.13, position = 116.6 cM) and chromosome 15 at D15S230 (LOD = 1.43, position = 135.6 cM) and D15S642 (LOD = 1.12, position = 152.1 cM). For the multipoint analysis, the only LOD score over 1.0 was 1.49, which occurred on chromosome 15 at 142 cM. Thus, in a direct comparison of the two-point screens using SNPs versus microsatellites, it is curious that higher LOD scores were detected with the SNPs than with the microsatellites, even though the SNPs are individually less informative. It may be the density of the SNP map that was beneficial and allowed the detection of linkage evidence that was missed by the microsatellite screen. To examine this hypothesis, we compared the locations of the trait-linked SNP markers on chromosomes 2 and 7 to the locations of the microsatellite markers using integrated map information from the National Center for Biotechnology Information (NCBI) [6], specifically, the NCBI build 35.1 reference assembly. This assembly places the peak SNP on chromosome 7, rs322812, at 127.338 Mb. This falls near the midpoint of the flanking microsatellites, which are located at 122.205 Mb and 131.736 Mb of the same build, indicating a modest gap, which is also borne out by the genetic positions of these microsatellites at 146.7 cM and 156.2 cM on the GAW14 map. However, for some of the other trait-linked SNPs, there do appear to be microsatellites positioned relatively close by. Other possible explanations for the higher LOD scores in the SNPs could be higher genotyping success rates in the SNPs or greater genotyping error in the microsatellites. The discrepancy could also be due to chance, for example if less informative SNPs resulted in some families being uninformative for linkage who might otherwise have contributed evidence against linkage.

Note also that in the microsatellite screens, the two-point analysis in many instances found higher LOD scores than the multipoint analysis. For example, the two-point analysis found weak evidence on chromosome 7 with a LOD score over 1 at 116.6 cM (in the vicinity of the SNP linkage signals), while multipoint LOD scores did not reach 1 on chromosome 7 (the maximum LOD was 0.89 at 140 cM).

## Conclusion

Our two-stage genome-wide analysis implicates chromosomes 2 and 7 as potentially harboring loci influencing the maximum drinks consumption phenotype. Follow-up association analysis supports chromosome 7 more strongly than chromosome 2, and suggests that further fine-mapping efforts on chromosome 7 may be constructive. Interestingly, this region of chromosome 7q contains several TAS2R bitter taste receptor genes [7], and haplotype status at one of these, TAS2R38, has been shown to be a significant predictor of alcohol intake [8]. The muscarinic receptor CHRM2 gene associated with alcohol dependence and major depressive syndrome in COGA [9] is also in this region.

Chromosome 4, which has been implicated in a previous linkage study of maximum drinks, did not pass our threshold for linkage evidence in the first stage of our primary analysis, but additional analyses of this chromosome indicated modest evidence for both linkage and association. In Saccone et al. [1] linkage to maximum drinks was found at marker D4S2407 on chromosome 4, which is located at 104.75 cM according to the Marshfield genetic map. Build 35.1 of the NCBI physical map puts this marker at approximately 97.60 Mb. This is near our linkage LOD score of 2.14 at rs1378876 located at 97.25 Mb on the same build (97.35 Mb on the GAW Illumina map). Hence, our supplementary analysis indicates some support for prior results on chromosome 4; however, our primary, two-stage analysis of the full genome does not support chromosome 4 and instead points to chromosome 7 as the strongest candidate for harboring a quantitative trait locus. The contrast between the chromosome 4 results for our primary analyses reported here and previous COGA findings is likely explained in part by the fact that the sample of families available for GAW14 is a subset of the full COGA dataset that was used in previously published COGA papers.

LD between markers is a concern when using multipoint linkage analysis, and our results show there is strong LD among SNPs on chromosome 7 which were found to be associated with maximum drinks. As an additional analysis we studied global LD structure using an algorithm we have previously described [10] to produce a non-overlapping set of blocks across all the chromosomes in the Illumina and Affymetrix SNP sets and found a significant amount of LD and variable block coverage on the different chromosomes, as expected. We are developing techniques for selecting "tag" SNPs from LD blocks which should be useful in this context by allowing maps to be thinned for multipoint linkage analysis, or by targeting "tag" SNPs for priority genotyping.

## Abbreviations

COGA: Collaborative Study on the Genetics of Alcoholism

GAW14: Genetic Analysis Workshop 14

LD: Linkage disequilibrium

MAF: Minor allele frequency

NCBI: National Center for Biotechnology Information

QPDT: Quantitative pedigree disequilibrium test

SNP: Single-nucleotide polymorphism

## Authors' contributions

SFS performed all statistical analyses and is the author of the program used to find LD blocks. All authors assisted in the design of the study and interpretation of results. In particular, NLS and JPR assisted in the design of the LD block program. SFS and NLS drafted the manuscript with input from all authors, and all authors read and approved the final manuscript.

## Figures and Tables

**Table 1:** Two-point linkage (TPL-stage 1) and trait association (QPDT-stage 2) results for chromosome 2

Marker	Source	MAF	Mbp	cM	Method	LOD	χ^2^	*p*-value
rs1025053	Illum	0.47	8.238	16.1	TPL	1.97	-	-
tsc0875114	Affy	0.21	8.345	21.7	QPDT	-^a^	9.05	0.003
rs1525351	Illum	0.44	216.466	212.9	TPL	2.68	-	-

^a^-, not applicable.

**Table 2:** Two-point linkage (stage 1) and trait association (QPDT-stage 2) results for chromosome 7

Marker	Source	MAF	Mbp	cM	Method	LOD	χ^2^	*p*-value
tsc0229102	Affy	0.46	89.250	101.9	QPDT	-^a^	7.29	0.007
rs727708	Illum	0.49	102.891	114.2	TPL	2.28	-	-
rs719530	Illum	0.42	109.451	121.8	TPL	2.14	-	-
rs846427	Illum	0.38	119.048	127.3	TPL	2.17	-	-
rs1860487	Illum	0.47	123.270	129.3	TPL	1.91	-	-
tsc0064426	Affy	0.37	124.757	128.6	QPDT	-	7.53	0.006
tsc1086900	Affy	0.37	124.758	128.6	QPDT	-	8.23	0.004
tsc0058943	Affy	0.42	124.791	128.6	QPDT	-	7.91	0.005
rs322812	Illum	0.44	127.299	131.3	TPL	2.77	-	-

^a^-, not applicable.

**Table 3:** Two-point regression (stage 1) and trait association (QPDT-stage 2) results for chromosome 4

Marker	Source	MAF	Mbp	cM	Method	LOD	χ^2^	*p*-value
tsc0592063	Affy	0.45	13.984	27.3	QPDT	-^a^	11.38	0.001
rs936232	Illum	0.42	25.564	37.1	REG	1.94	-	-
rs1031739	Illum	0.40	87.688	93.1	QPDT	-	6.99	0.008
rs2046015	Illum	0.43	94.144	98.1	QPDT	-	7.47	0.006
rs0965823	Affy	0.49	96.436	102.3	QPDT	-	4.35	0.037
rs1378876	Illum	0.37	97.354	100.4	REG	2.14	-	-
rs960345	Illum	0.46	105.689	105.6	QPDT	-	8.50	0.004
rs0724156	Affy	0.24	131.384	128.1	QPDT	-	8.62	0.003
rs1365372	Illum	0.12	139.914	129.8	REG	1.91	-	-

^a^-, not applicable.

## References

[B1] Saccone NL, Kwon JM, Corbett J, Goate A, Rochberg N, Edenberg HJ, Foroud T, Li TK, Begleiter H, Reich T, Rice JP (2000). A genome screen of the maximum number of drinks as an alcoholism phenotype. Am J Med Genet.

[B2] Abecasis GR, Cherny SS, Cookson WO, Cardon LR (2001). Merlin-rapid analysis of genetic maps using sparse gene flow trees. Nat Genet.

[B3] Monks SA, Kaplan NL (2000). Removing the sampling restriction from family-based tests of association for a quantitative-trait locus. Am J Hum Genet.

[B4] Dudbridge F (2003). Pedigree disequilibrium test for multilocus haplotypes. Genet Epidemiol.

[B5] Sham PC, Purcell S, Cherny S, Abecasis R (2002). Powerful regression-based quantitative-trait linkage analysis of general pedigrees. Am J Hum Genet.

[B6] Abecasis GR, Cookson WOC (2000). GOLD-Graphical overview of linkage disequilibrium. Bioinformatics.

[B7] Adler E, Hoon MA, Mueller KL, Chandrashekar J, Ryba NJP, Zuker CS (2000). A novel family of mammalian taste receptors. Cell.

[B8] Duffy VB, Davidson AC, Kidd JR, Kidd KK, Speed WC, Pakstis AJ, Reed DR, Snyder DJ, Bartoshuk LM (2004). Bitter receptor gene (TAS2R38), 6-n-probylthiouracil (PROP) bitterness and alcohol intake. Alcohol Clin Exp Res.

[B9] Wang JC, Hinrichs AL, Stock H, Budde J, Allen R, Bertelsen S, Kwon JM, Wu W, Dick DM, Jones K, Nurnberger JI, Tischfield J, Porjesz B, Edenberg HJ, Hesselbrock V, Crowe R, Schuckit M, Begleiter H, Reich T, Goate AM, Bierut LJ (2004). Evidence of common and specific genetic effects: association of the muscarinic acetylcholine receptor M2 (CHRM2) gene with alcohol dependence and major depressive syndrome. Hum Mol Genet.

[B10] Taillon-Miller P, Saccone SF, Saccone NL, Duan S, Kloss EF, Lovins EG, Donaldson R, Phong A, Ha C, Flagstad L, Miller S, Drendel A, Lind D, Miller RD, Rice JP, Kwok P-Y (2004). Linkage disequilibrium maps constructed with common SNPs are useful for first-pass disease association screens. Genomics.

